# Supplementary material for: Uncovering temporal changes in Europe’s population density patterns using a data fusion approach
Source: Nat Commun. 2020 Sep 15;11:4631. doi: 10.1038/s41467-020-18344-5 (PMC7493994; doi:10.1038/s41467-020-18344-5)
Supplement: Supplementary file 1 — Supplementary Information [file 41467_2020_18344_MOESM1_ESM.pdf]

Supplementary Information for

Uncovering temporal changes in Europe's population density patterns using a data fusion approach

Batista e Silva* *et al.*

*Corresponding author. Email: filipe.batista@ec.europa.eu

Supplementary Note 1. Description of key input datasets used for the production of the multitemporal population grids

The European Settlement Map (ESM) is a spatial raster dataset mapping human settlements in Europe. It is based on SPOT5 and SPOT6 satellite imagery at Very High Resolution and on machine learning techniques to understand systematic relations between morphological and textural (pantex) features typical of human settlements[1].

The ESM – release 2017 is a raster layer with a native resolution of 2.5 m (same as the input Very High Resolution images) classifying pixels as buildings, green areas, open spaces, streets, railways and water by integrating satellite processed images with information from other available sources (Urban Atlas, Tele Atlas, Open Street Map and National Datasets)[2].

The ESM dataset is publicly available in two aggregated resolutions (10 m and 100 m) each composed of 13 raster layers, one for each land cover class. The values of the pixels in each layer represent the proportion of the respective land cover class within the pixel. In this study we used only the built-up layer at 100 m resolution as reference for the horizontal density of buildings, i.e. the percentage of surface covered by all roofed constructions, and varying from 0 to 100%.

The land use/land cover (LULC) map was produced by integrating geospatial data from a wealth of sources. It is originally based on the CORINE Land Cover (CLC) 2012 map and nomenclature (https://land.copernicus.eu/pan-european/corine-land-cover), but achieves superior spatial and thematic detail. The spatial detail was increased by merging CLC, European Settlement Map built-up layer, Urban Atlas, Copernicus high-resolution layers and other geodata. The original minimum mapping unit (MMU) of 25 ha was reduced significantly, depending on the data-source applied and LULC type. Given the purpose of the map, and allowed by a higher detail of the related data sources, artificial land cover patches as small as 1 ha were included, whereas 5 ha level was deemed adequate for the refinement of other classes.

In the process, new classes were derived using spatial data on building footprints. Urban fabric class was subdivided into four distinct density bands. Furthermore, the class ‘sport and leisure facilities’ was split into built-up and green components, airport terminals were extracted out of the airport class and major stations were extracted from the ground transportation class. Consequently, a machine-learning approach was used to breakdown the general ‘industry/commerce’ class into more detailed classes (‘production’, ‘commerce and services’, and ‘public facilities’), based on the presence or absence of several categories of Points Of Interest. The expansion of 11 artificial LULC classes into 18 was instrumental for the allocation of certain population groups. The resulting map covers the EU-27 and other European countries and contains 50 classes in total. The production and validation of this novel map has been documented in a dedicated article[3].

With regards to the use of Point of Interest (POI) data, it was assumed that OpenStreetMap and TomTom are the most complete and accurate sources covering the entire territory of Europe. Although not perfect, omitting any or both of these sources would forbid the method we chose.

Unfortunately, there is no available literature on the quality of TomTom. TomTom MultiNet is a proprietary off-the-shelf dataset intended for satellite navigation systems; in multiple studies with European focus, it was actually used as a benchmark to evaluate the completeness of volunteered data[4,5].

As for the quality of the OSM data, several studies suggest that despite being collected by volunteers, it has a great value and should not be overlooked[6,7] and attempts were made to produce LULC maps[8,9] and population maps[10] based on OSM POI.

As for the temporal relation to the population grids: the 2011 census preceded the collection/delivery of the used datasets by several years. In our application, the datasets can contain features added as late as 2017. However, due to lack of exact temporal validity information in the data, it is hard to approach this in a

better way than just including all of the available features. Although it would be possible to exclude objects added after 2011 using the timestamp, this would discard the great majority of OSM POI (see chart below). The database completeness has been growing significantly in the period 2011-2017 due to increasing popularity of OpenStreetMap, rather than due to new facilities in existence.

The situation is similar for TomTom data – the database is periodically updated, but it is not feasible to distinguish the reason behind new POI additions.

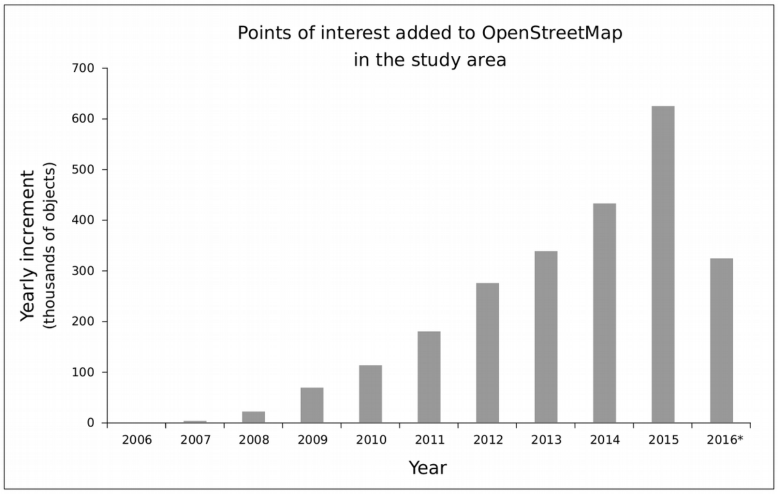

Supplementary Figure 1. Yearly increments of POI-type objects in OpenStreeMap, based on a data-dump from 16/06/2016.

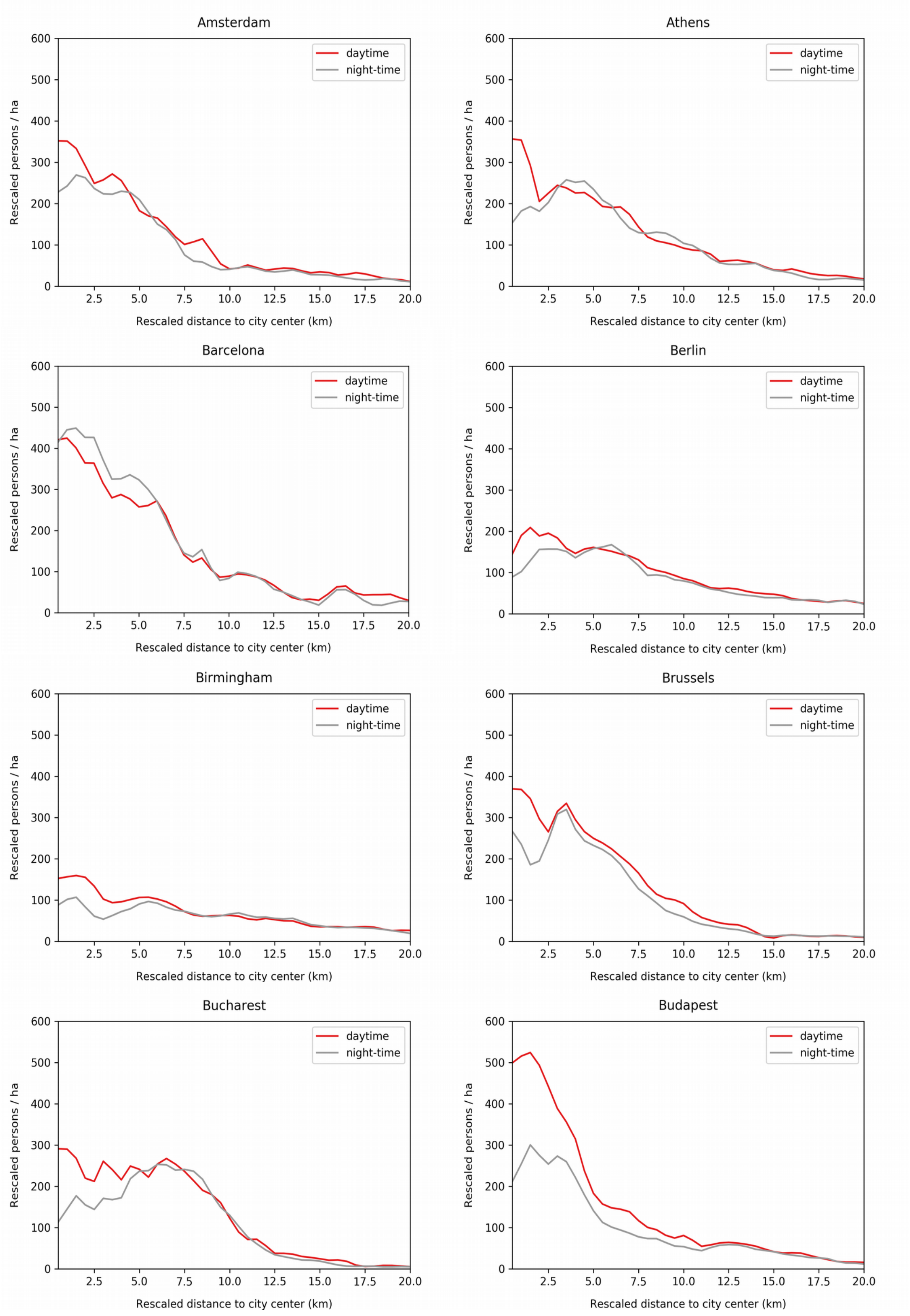

Supplementary Figure 2. Day- and night-time concentric population density profiles for cities with names starting from letters A to B. Population densities and distance to city center were rescaled as prescribed by Lemoy and Caruso[11] to make the population density profiles comparable across cities of different population sizes. Source data are provided as a Source Data spreadsheet file.

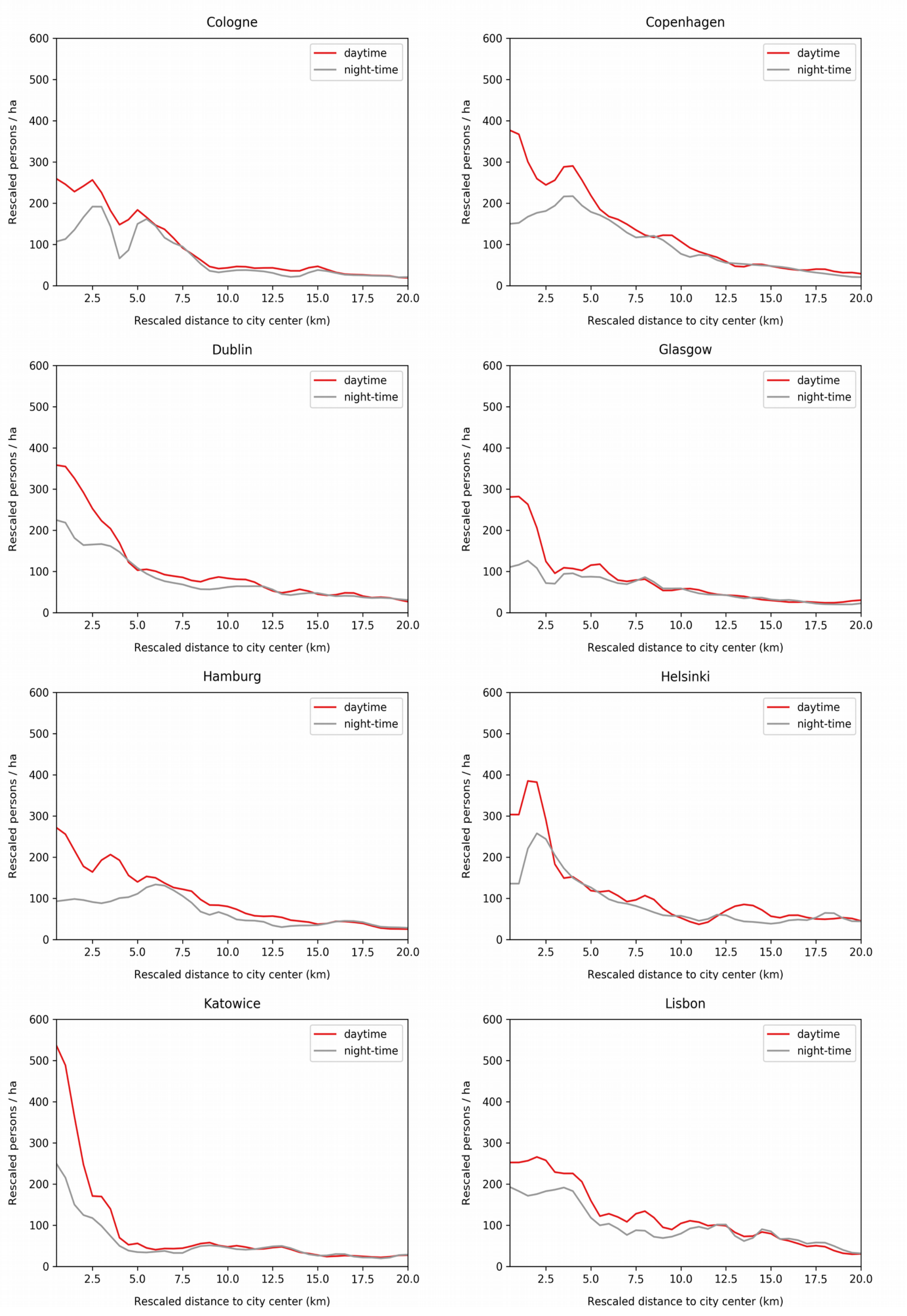

Supplementary Figure 3. Day- and night-time concentric population density profiles for cities with names starting from letters C to Lis. Population densities and distance to city center were rescaled as prescribed by Lemoy and Caruso[11] to make the population density profiles comparable across cities of different population sizes. Source data are provided as a Source Data spreadsheet file.

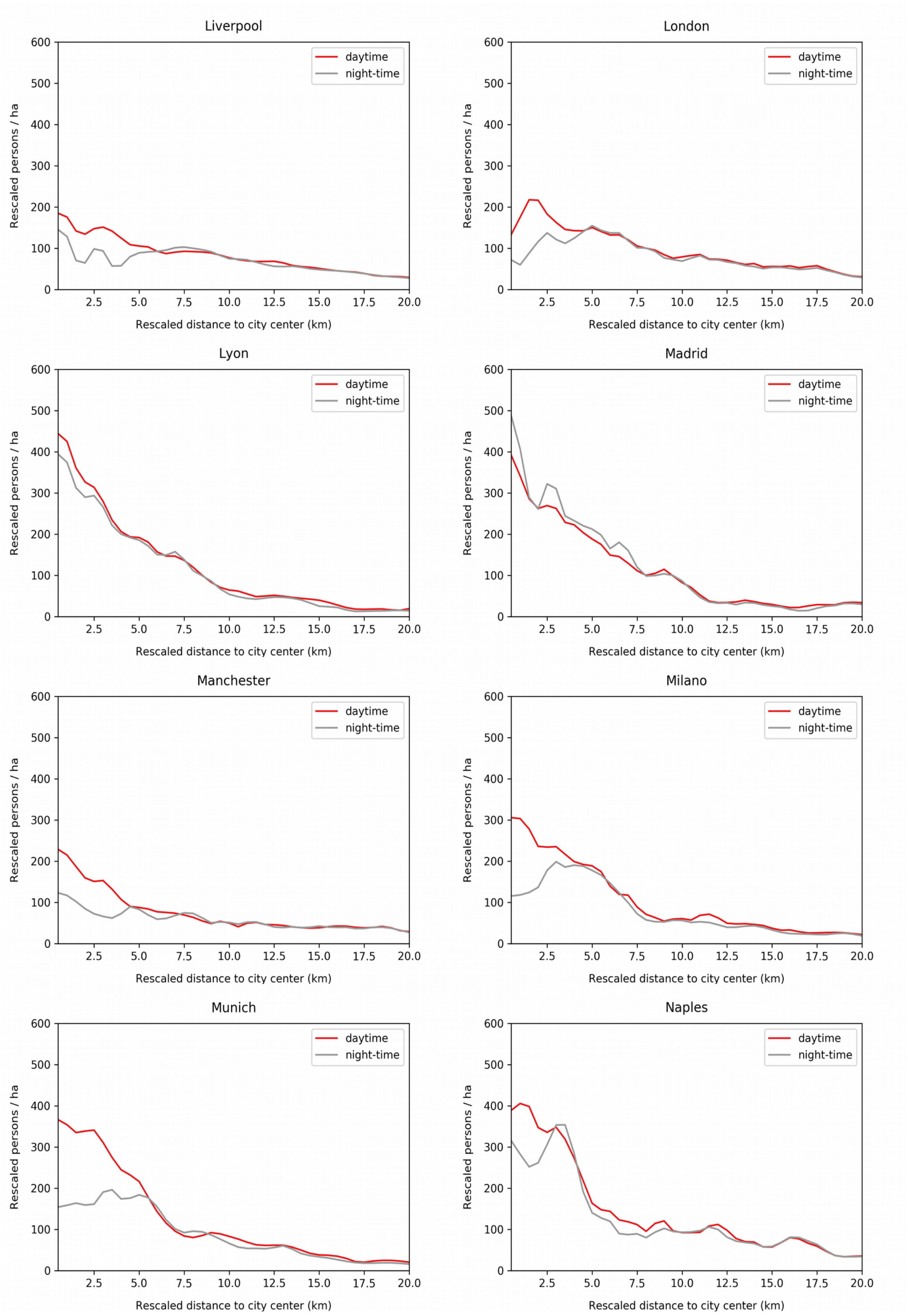

Supplementary Figure 4. Day- and night-time concentric population density profiles for cities with names starting from letters Liv to N. Population densities and distance to city center were rescaled as prescribed by Lemoy and Caruso[11] to make the population density profiles comparable across cities of different population sizes. Source data are provided as a Source Data spreadsheet file.

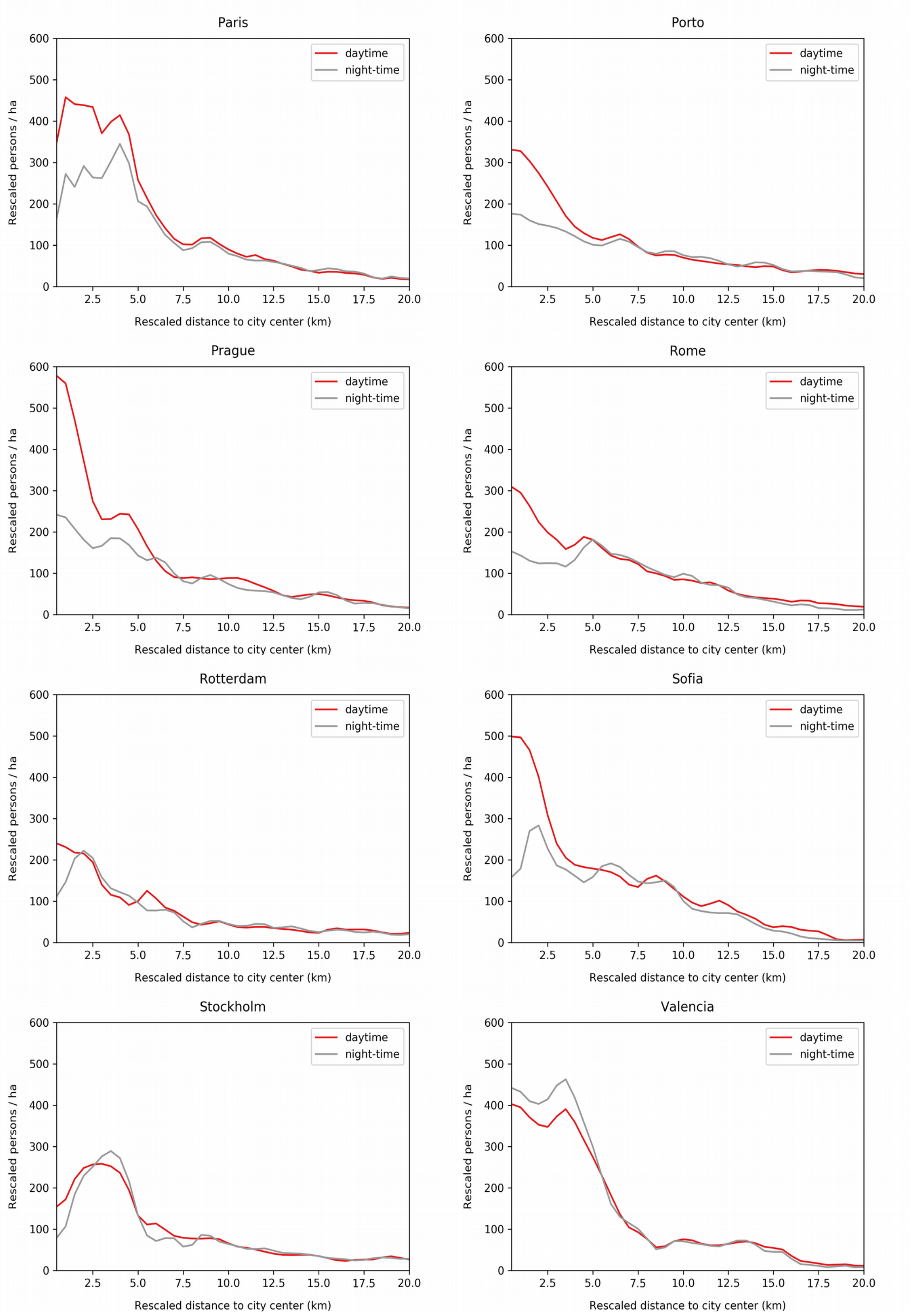

Supplementary Figure 5. Day- and night-time concrentric population density profiles for cities with names starting from letters P to Va. Population densities and distance to city center were rescaled as prescribed by Lemoy and Caruso[11] to make the population density profiles comparable across cities of different population sizes. Source data are provided as a Source Data spreadsheet file.

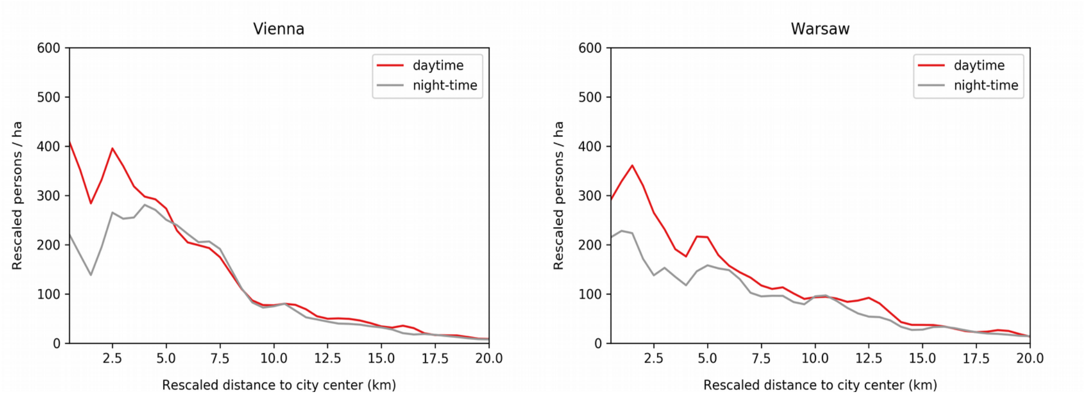

Supplementary Figure 6. Day- and night-time concentric population density profiles for cities with names starting from letters Vi to W. Population densities and distance to city center were rescaled as prescribed by Lemoy and Caruso[11] to make the population density profiles comparable across cities of different population sizes. Source data are provided as a Source Data spreadsheet file.

Supplementary Table 1. Summary statistics for sampled cities/greater cities, based on yearly average. Ordered from largest to smallest city based on night-time population.

**City**	**Country**	**Area (km^2)**	**Population**			**Population density**		**Employees**		**Students**		**Tourists**	
			Daytime	Night-time	Ratio	Daytime	Night-time	Abs.	Rel. (%)	Abs.	Rel. (%)	Abs.	Rel. (%)
**Paris**	FR	1,910	10,494,728	9,635,775	1.089	5,496	5,046	5,160,407	49.2	2,387,529	22.7	153,215	1.5
**London**	UK	1,576	8,624,184	8,119,979	1.062	5,473	5,153	4,427,000	51.3	1,863,942	21.6	163,969	1.9
**Madrid**	ES	1,232	4,840,136	4,916,371	0.984	3,928	3,990	2,220,984	45.9	1,055,230	21.8	58,665	1.2
**Milan**	IT	1,983	4,404,378	3,843,081	1.146	2,221	1,938	2,161,600	49.1	750,473	17.0	26,929	0.6
**Barcelona**	ES	599	3,460,856	3,634,273	0.952	5,780	6,070	1,415,573	40.9	757,647	21.9	39,404	1.1
**Athens**	EL	613	3,269,311	3,304,539	0.989	5,335	5,393	1,212,185	37.1	669,371	20.5	14,859	0.5
**Berlin**	DE	892	3,425,093	3,262,189	1.05	3,840	3,658	1,707,610	49.9	645,856	18.9	84,518	2.5
**Naples**	IT	1,173	3,261,838	3,030,807	1.076	2,780	2,583	989,100	30.3	748,328	22.9	22,534	0.7
**Manchester**	UK	1,277	2,681,058	2,630,894	1.019	2,100	2,060	1,281,000	47.8	639,244	23.8	16,597	0.6
**Romes**	IT	1,286	2,965,024	2,622,010	1.131	2,306	2,039	1,280,687	43.2	660,648	22.3	48,781	1.6
**Birmingham**	UK	804	2,389,343	2,373,188	1.007	2,973	2,953	1,059,000	44.3	592,786	24.8	14,792	0.6
**Katowice**	PL	1,218	1,996,743	1,920,611	1.04	1,639	1,577	848,213	42.5	429,650	21.5	3,709	0.2
**Lisbon**	PT	637	2,032,627	1,878,655	1.082	3,191	2,949	952,583	46.9	456,936	22.5	32,144	1.6
**Bucharest**	RO	240	2,010,562	1,874,175	1.073	8,392	7,823	982,358	48.9	527,750	26.2	8,441	0.4
**Budapest**	HU	525	2,273,490	1,729,545	1.315	4,329	3,293	1,261,862	55.5	397,610	17.5	21,505	0.9
**Warsaw**	PL	517	2,225,749	1,698,340	1.311	4,303	3,284	1,145,654	51.5	595,940	26.8	12,412	0.6
**Vienna**	AT	415	1,884,473	1,696,604	1.111	4,543	4,090	987,342	52.4	437,965	23.2	40,401	2.1
**Hamburg**	DE	739	1,973,961	1,686,044	1.171	2,670	2,281	1,148,538	58.2	364,368	18.5	36,483	1.8
**Stockholm**	SE	1,391	1,507,647	1,585,891	0.951	1,084	1,140	855,011	56.7	432,442	28.7	27,947	1.9
**Valencia**	ES	401	1,313,862	1,400,391	0.938	3,277	3,493	419,248	31.9	333,931	25.4	19,190	1.5
**Liverpool**	UK	646	1,389,325	1,356,118	1.024	2,152	2,100	628,055	45.2	307,414	22.1	9,445	0.7
**Munich**	DE	311	1,614,002	1,330,346	1.213	5,183	4,272	982,020	60.8	282,786	17.5	24,212	1.5
**Prague**	CZ	496	1,531,018	1,289,667	1.187	3,084	2,598	900,000	58.8	363,112	23.7	45,889	3.0
**Dublin**	IE	926	1,389,508	1,250,085	1.112	1,501	1,351	648,400	46.7	340,037	24.5	22,519	1.6
**Sofia**	BG	450	1,460,780	1,196,820	1.221	3,246	2,659	794,992	54.4	261,982	17.9	3,624	0.2
**Rotterdam**	NL	563	1,182,625	1,151,860	1.027	2,102	2,048	625,409	52.9	283,340	24.0	8,491	0.7
**Brussels**	BE	162	1,395,435	1,124,738	1.241	8,589	6,923	688,900	49.4	393,760	28.2	14,268	1.0
**Copenhagen**	DK	457	1,282,197	1,114,436	1.151	2,805	2,438	727,940	56.8	323,297	25.2	15,857	1.2
**Helsinki**	FI	779	1,052,832	1,031,149	1.021	1,351	1,323	561,479	53.3	281,892	26.8	12,315	1.2
**Lyon**	FR	220	1,083,108	1,008,056	1.074	4,919	4,578	477,600	44.1	279,153	25.8	5,991	0.6
**Cologne**	DE	407	1,217,080	990,355	1.229	2,992	2,435	687,245	56.5	234,775	19.3	12,103	1.0
**Porto**	PT	479	1,014,321	972,146	1.043	2,116	2,028	474,522	46.8	226,924	22.4	9,484	0.9
**Glasgow**	UK	786	997,052	944,557	1.056	1,268	1,201	484,802	48.6	206,492	20.7	6,851	0.7
**Amsterdam**	NL	303	1,075,723	888,080	1.211	3,546	2,927	605,118	56.3	259,508	24.1	26,486	2.5
**Minimum**		162			0.938	1,084	1,140		30.3		17.0		0.2
**Maximum**		1,983			1.315	8,589	7,823		60.8		28.7		3.0
**Average**		777			1.097	3,545	3,226		48.9		22.7		1.2
**Standard dev.**		464			0.098	1,799	1,622		7.0		3.0		0.7

Supplementary Table 2. Correspondence between land use classes and POI-based activity layers with population groups for day- and night-time population mapping.

Land use class / POI activity layer	Night-time		Day-time														
	Residents	Tourists	Employees											Students		Non-working, non-studying	Tourists
			‘A’	‘BDE’	‘C’	‘F’	‘GHI’	‘J’	‘K’	‘L’	‘MN’	‘OPQ’	‘RSTU’	‘04’	‘56’		
CLCr_1111	1	0	0	0	0	1	0	0	0	0	0	0	1	0	0	1	0
CLCr_1121	1	0	0	0	0	1	0	0	0	0	0	0	1	0	1	1	0
CLCr_1122	1	0	0	0	0	1	0	0	0	0	0	0	1	0	1	1	0
CLCr_1123	1	0	0	0	0	1	0	0	0	0	0	0	1	0	1	1	0
CLCr_1211	0	0	1	1	1	1	0	0	0	0	0	0	0	0	0	0	0
CLCr_1212	0	0	0	0	0	1	1	1	1	1	1	0	1	0	0	0	1
CLCr_1213	0	0	0	0	0	1	0	0	0	0	0	1	1	0	0	0	1
CLCr_1222	0	0	0	0	0	1	1	0	0	0	0	0	0	0	0	0	1
CLCr_1231	0	0	1	0	0	1	1	0	0	0	0	0	0	0	0	0	1
CLCr_1242	0	0	0	0	0	1	1	0	0	0	0	1	0	0	0	0	1
CLCr_1311	0	0	0	1	0	0	0	0	0	0	0	0	0	0	0	0	0
CLCr_1321	0	0	0	1	0	0	0	0	0	0	0	0	0	0	0	0	0
CLCr_1331	0	0	0	0	0	1	0	0	0	0	0	0	0	0	0	0	0
CLCr_1411	0	0	0	0	0	0	0	0	0	0	0	0	0	0	0	0	1
CLCr_1421	0	0	0	0	0	0	0	0	0	0	0	0	0	0	0	0	1
CLCr_1422	0	0	0	0	0	1	1	0	0	0	0	0	1	0	0	0	1
POI_A	0	0	1	0	0	0	0	0	0	0	0	0	0	0	0	0	0
POI_BDE	0	0	0	1	0	0	0	0	0	0	0	0	0	0	0	0	0
POI_C	0	0	0	0	1	0	0	0	0	0	0	0	0	0	0	0	0
POI_F	0	0	0	0	0	1	0	0	0	0	0	0	0	0	0	0	0
POI_GHI	0	0	0	0	0	0	1	0	0	0	0	0	0	0	0	0	1
POI_JKL	0	0	0	0	0	0	0	1	1	1	0	0	0	0	0	0	1
POI_MN	0	0	0	0	0	0	0	0	0	0	1	0	0	0	0	0	0
POI_OPQ	0	0	0	0	0	0	0	0	0	0	0	1	0	0	0	0	0
POI_RSTU	0	0	0	0	0	0	0	0	0	0	0	0	1	0	0	0	1
POI_Tour	0	1	0	0	0	0	0	0	0	0	0	0	0	0	0	0	1
POI_S04	0	0	0	0	0	0	0	0	0	0	0	0	0	1	0	0	0
POI_S56	0	0	0	0	0	0	0	0	0	0	0	0	0	0	1	0	0

Supplementary Table 3. List and description of land use classes from the LULC map and POI-based activity layers.

**Land use class / POI activity layer**	**Description**
CLCr_1111	Urban fabric dense
CLCr_1121	Urban fabric medium density
CLCr_1122	Urban fabric low density
CLCr_1123	Urban fabric very low density / isolated
CLCr_1211	Production facilities
CLCr_1212	Commercial & service facilities
CLCr_1213	Public facilities
CLCr_1222	Rail stations
CLCr_1231	Port areas
CLCr_1242	Airport terminals
CLCr_1311	Mineral extraction sites
CLCr_1321	Dump sites
CLCr_1331	Construction sites
CLCr_1411	Green urban areas
CLCr_1421	Sport and leisure green
CLCr_1422	Sport, leisure and touristic built-up
POI_A	Points of Interest related to NACE sector A
POI_BDE	Points of Interest related to NACE sectors B, D and E
POI_C	Points of Interest related to NACE sector C
POI_F	Points of Interest related to NACE sector F
POI_GHI	Points of Interest related to NACE sectors G, H and I
POI_JKL	Points of Interest related to NACE sectors J, K and L
POI_MN	Points of Interest related to NACE sectors M and N
POI_OPQ	Points of Interest related to NACE sectors O, P and Q
POI_RSTU	Points of Interest related to NACE sectors R, S, T, and U
POI_Tour	Touristic accommodation room density
POI_S04	Locations of schools
POI_S56	Locations of universities

Supplementary Table 4. List and description of NACE rev. 2 categories used in the study.

NACE category	Description
A	Agriculture, forestry and fishing
BDE	Industry (except construction)
C	Manufacturing
F	Construction
GHI	Wholesale and retail trade, transport, accommodation and food service activities
J	Information and communication
K	Financial and insurance activities
L	Real estate activities
MN	Professional, scientific and technical activities; administrative and support service activities
OPQ	Public administration, defense, education, human health and social work activities
RSTU	Arts, entertainment and recreation; other service activities; activities of household and extra-territorial organizations and bodies

Supplementary References

1. Florczyk, A. J. *et al.* A New European Settlement Map From Optical Remotely Sensed Data. *IEEE J. Sel. Top. Appl. Earth Obs. Remote Sens.* **9**, 1978–1992 (2016).
2. Ferri, S., Siragura, A., Sabo, F., Pafi, M. & Halkia, M. *The European Settlement Map - 2017 release*. (2017). doi:10.2760/780799
3. Rosina, K. *et al.* Increasing the detail of European land use/cover data by combining heterogeneous data sets. *Int. J. Digit. Earth* **13**, 602–626 (2020).
4. Zielstra, D. & Zipf, A. A comparative study of proprietary geodata and volunteered geographic information for Germany. in *Proceedings of the Thirteenth AGILE International Conference on Geographic Information Science* (2010).
5. Neis, P., Zielstra, D. & Zipf, A. The Street Network Evolution of Crowdsourced Maps: OpenStreetMap in Germany 2007–2011. *Futur. Internet* **4**, 1–21 (2012).
6. Dorn, H., Törnros, T. & Zipf, A. Quality evaluation of VGI using authoritative data-a comparison with land use data in southern Germany. *ISPRS Int. J. Geo-Information* **4**, 1657–1671 (2015).
7. Jokar Arsanjani, J. & Vaz, E. An assessment of a collaborative mapping approach for exploring land use patterns for several European metropolises. *Int. J. Appl. Earth Obs. Geoinf.* **35**, 329–337 (2015).
8. Jokar Arsanjani, J., Helbich, M., Bakillah, M., Hagenauer, J. & Zipf, A. Toward mapping land-use patterns from volunteered geographic information. *Int. J. Geogr. Inf. Sci.* **27**, 2264–2278 (2013).
9. Estima, J. & Painho, A. Investigating the Potential of OpenStreetMap for Land Use/Land Cover Production: A Case Study for Continental Portugal. in *OpenStreetMap in GIScience. Lecture Notes in Geoinformation and Cartography.* (eds. Jokar Arsanjani, J., Zipf, A., Mooney, P. & Helbich, M.) 273–293 (Springer International Publishing, 2015).

10. Bakillah, M., Liang, S., Mobasheri, A., Jokar Arsanjani, J. & Zipf, A. Fine-resolution population mapping using OpenStreetMap points-of-interest. *Int. J. Geogr. Inf. Sci.* **28**, 1940–1963 (2014).

11. Lemoy, R. & Caruso, G. Evidence for the homothetic scaling of urban forms. *Environ. Plan. B Urban Anal. City Sci.* (2018). doi:10.1177/2399808318810532
