# Supplementary material for: Uncovering temporal changes in Europe’s population density patterns using a data fusion approach
Source: Nat Commun. 2020 Sep 15;11:4631. doi: 10.1038/s41467-020-18344-5 (PMC7493994; doi:10.1038/s41467-020-18344-5)
Supplement: Supplementary file 3 — Reporting Summary [file 41467_2020_18344_MOESM3_ESM.pdf]

nature research

Corresponding author(s): Filipe Batista e Silva

Last updated by author(s): May 11, 2020

Reporting Summary

Nature Research wishes to improve the reproducibility of the work that we publish. This form provides structure for consistency and transparency in reporting. For further information on Nature Research policies, see our Editorial Policies and the Editorial Policy Checklist.

Statistics

For all statistical analyses, confirm that the following items are present in the figure legend, table legend, main text, or Methods section.

n/a	Confirmed	
☐	☒	The exact sample size (n) for each experimental group/condition, given as a discrete number and unit of measurement
☒	☐	A statement on whether measurements were taken from distinct samples or whether the same sample was measured repeatedly
☒	☐	The statistical test(s) used AND whether they are one- or two-sided<br>*Only common tests should be described solely by name; describe more complex techniques in the Methods section.*
☐	☒	A description of all covariates tested
☐	☒	A description of any assumptions or corrections, such as tests of normality and adjustment for multiple comparisons
☐	☒	A full description of the statistical parameters including central tendency (e.g. means) or other basic estimates (e.g. regression coefficient) AND variation (e.g. standard deviation) or associated estimates of uncertainty (e.g. confidence intervals)
☒	☐	For null hypothesis testing, the test statistic (e.g. F, t, r) with confidence intervals, effect sizes, degrees of freedom and P value noted<br>*Give P values as exact values whenever suitable.*
☒	☐	For Bayesian analysis, information on the choice of priors and Markov chain Monte Carlo settings
☒	☐	For hierarchical and complex designs, identification of the appropriate level for tests and full reporting of outcomes
☒	☐	Estimates of effect sizes (e.g. Cohen's d, Pearson's r), indicating how they were calculated

Our web collection on statistics for biologists contains articles on many of the points above.

Software and code

Policy information about availability of computer code

Data collection	No specialized software was used to collect the input data used this study. The statistical and geospatial data used in our study were obtained from the indicated sources in the manuscript, usually by downloading directly from the sources online repositories or via institutional agreements (only applicable to data from TomTom and Proximus).
Data analysis	The estimation of monthly and regional population stocks and the cross-comparison were done primarily using Excel spreadsheets. The pre-processing and analysis of geospatial data were peformed using ESRI ArcGIS 10 and Python 2 using arcpy library. The dasymetric modelling was implemented in Matlab 9. The analysis of the results (spatio-temporal city characteristics) was peformed using Python 3 using pandas and sklearn libraries. The map visualizations were performed using QGIS 3 (Figure 1) ERSI ArcGIS 10 (Figure 2). Any potentially useful code produced can be made available upon reasonable request.

For manuscripts utilizing custom algorithms or software that are central to the research but not yet described in published literature, software must be made available to editors and reviewers. We strongly encourage code deposition in a community repository (e.g. GitHub). See the Nature Research guidelines for submitting code & software for further information.

Data

Policy information about availability of data

All manuscripts must include a data availability statement. This statement should provide the following information, where applicable:

- Accession codes, unique identifiers, or web links for publicly available datasets
- A list of figures that have associated raw data
- A description of any restrictions on data availability

The multitemporal population grids for the European Union at 1 km2 resolution that have been generated during this study75 have been deposited in the European Commission's Joint Research Centre Data Catalogue, with identifier doi:10.2905/BE02937C-5A08-4732-A24A-03E0A48BDCDA, and can be accessed at https://

data.jrc.ec.europa.eu/dataset/be02937c-5a08-4732-a24a-03e0a48bdcda. These multitemporal grids are the source data for Figs 1 and 2. The source data underlying Figs 3a-c and 4, and Supplementary Figs 2-6 are provided as a Source Data file.

Field-specific reporting

Please select the one below that is the best fit for your research. If you are not sure, read the appropriate sections before making your selection.

☐ Life sciences ☐ Behavioural & social sciences ☒ Ecological, evolutionary & environmental sciences

For a reference copy of the document with all sections, see nature.com/documents/nr-reporting-summary-flat.pdf

Ecological, evolutionary & environmental sciences study design

All studies must disclose on these points even when the disclosure is negative.

Study description	In this study we produced a novel dataset of multitemporal population grids at a spatial resolution of 1 km x 1 km, capturing both intraday and seasonal population variations for the European Union (EU). The dataset is composed of 24 individual raster grid files covering the EU in a seamless fashion. The dataset was produced by combining two main data types: official statistics on population groups per subnational zoning systems and geospatial covariates of those population groups. The data combination was done following a novel elaboration of the 'dasymetric approach'. To illustrate the relevance, potential as well as the validity of the produced dataset we carried an analysis to a sample of the N=34 largest cities within the study area, focusing on the spatio-temporal structure of cities. This analysis is mostly of descriptive nature (including descriptive statistics, plotting and cluster analysis). No hypothesis testing was performed.
Research sample	European Union cities.
Sampling strategy	To select the largest European Union cities, we used the city/greater city extents defined by Eurostat. This definition was designed to improve comparability of city statistics, and applies a fixed set of criteria related to urban morphology to consistently characterize city limits irrespective of national definitions. For each of the listed 800+ cities/greater cities, we summed the population in day- and night-time based on yearly average grids, and selected those whose day- or night-time population is above 1 million people, resulting in a sample size of N = 34.
Data collection	Statistical and geospatial data collection was peformed by various co-authors, namely F.B.S., M.S., K.R. and M.A.M.H. The data were downloaded from open statistical and mapping online repositories (e.g. Eurostat, National Statistical Offices, Copernicus, OpenStreetMap). The TomTom geospatial and the mobile phone records used for the cross-comparison were obtained via institutional agreement. All data were obtained during the research project duration (2016-2018).
Timing and spatial scale	The statistical and geospatial data were collected during the research project duration (2016-2018). Demographic variables refer to the year 2011 (demographic variables), land use to 2012 and Points of Interest to 2017. Statistical data was obtained at subnational level ('NUTS3' units from the European Statistical System), and geospatial data were obtained at a spatial resolution of 100x100 m or smaller.
Data exclusions	Our study mapped spatio-temporal population for the European Union 28 countries (European Union as defined as of 2019, therefore including U.K.). The analysis in the resuts section was performed to a sample of the 34 largest cities within the referred area of interest. There were no data exclusions within the area of interest.
Reproducibility	The produced dataset can be reproduced by applying the same input data and data fusion procedures, equations and parameters described in detail in the methods section of the manuscript. Our main concern was to provide the most detailed description possible of the data fusion procedures and input data. Even so, any code produced can be made available upon reasonable request to the authors.
Randomization	Not applicable. Our study was about producing a detailed dataset of spatio-temporal population and analysing the results.
Blinding	Not applicable. Our study was about producing a detailed dataset of spatio-temporal population and analysing the results.

Did the study involve field work? ☐ Yes ☒ No

Reporting for specific materials, systems and methods

We require information from authors about some types of materials, experimental systems and methods used in many studies. Here, indicate whether each material, system or method listed is relevant to your study. If you are not sure if a list item applies to your research, read the appropriate section before selecting a response.

Materials & experimental systems

n/a	Involved in the study
☒	☐ Antibodies
☒	☐ Eukaryotic cell lines
☒	☐ Palaeontology and archaeology
☒	☐ Animals and other organisms
☒	☐ Human research participants
☒	☐ Clinical data
☒	☐ Dual use research of concern

Methods

n/a	Involved in the study
☒	☐ ChIP-seq
☒	☐ Flow cytometry
☒	☐ MRI-based neuroimaging
